# Multidisciplinary rehabilitation with a focus on physiotherapy in patients with Post Covid19 condition: an observational pilot study

**DOI:** 10.1007/s00406-023-01747-y

**Published:** 2024-01-17

**Authors:** Martin Weigl, Saskia Beeck, Eduard Kraft, Hans Christian Stubbe, Kristina Adorjan, Michael Ruzicka, Christina Lemhöfer

**Affiliations:** 1 Department of Orthopaedics and Trauma Surgery, Physical and Rehabilitation Medicine, Musculoskeletal University Center Munich (MUM), University Hospital, LMU Munich, Marchioninistr. 15, 81377 Munich, Germany; 2 Munich Municipal Hospital Group, Munich, Germany; 3 Department of Psychiatry and Psychotherapy, LMU University Hospital Munich, Munich, Germany; 4 Department of Medicine II, LMU University Hospital Munich, Munich, Germany; 5 Department of Medicine III, University Hospital, LMU Munich, Munich, Germany; 6 Institute of Physical and Rehabilitation Medicine, Jena University Hospital, Jena, Germany

**Keywords:** Post-COVID condition, Rehabilitation, Physiotherapy, Exercise therapy, Breathing exercises

## Abstract

There is a lack of interventions that treat the Post-Covid-19 Condition (PCC) itself. Accordingly, treatment guidelines recommend physiotherapy interventions to alleviate symptoms and enhance functioning. In cases where unimodal treatments prove ineffective, non-organ-specific multidisciplinary bio-psycho-social rehabilitation (MBR) programs are a suitable option. In a pilot observational study with assessments at the entry and end of treatment we aimed to evaluate the feasibility of a 3-week day clinic MBR program and explore its effects on physical functioning in PCC patients with fatigue and reduced physical capacity. Patient selection was based on an interdisciplinary assessment involving a physician, a psychologist and a physiotherapist. Feasibility was determined based on full participation (≥ 8 of 9 days) and maintenance of stable endurance in the 6-Minute Walk Test (6MWT). From 37 patients included in the study, 33 completed the MBR (mean age: 43 ± 12 years, 73% female). Four patients discontinued the MBR, with two of them having reported deterioration of PCC symptoms. The 6MWT showed a numerical improvement from 501 ± 97 m to 512 ± 87 m, although it did not reach statistical significance. These results support the feasibility of outpatient MBR with a focus on active physiotherapy interventions in PCC patients with fatigue. This study aligns with previous research supporting the effectiveness of physiotherapy and rehabilitation in PCC patients. However, further research is needed to address possible different treatment responses and varying treatment approaches in subgroups of PCC patients.

**Supplementary Information:**

The online version contains supplementary material available at 10.1007/s00406-023-01747-y.

## Introduction

The World Health Organization (WHO) defines the Post-Covid-19 condition (PCC) “as the continuation or development of new symptoms 3 months after the initial SARS-CoV-2 infection, with these symptoms lasting for at least 2 months with no other explanation [1]. Common symptoms in patients with PCC are fatigue, dyspnoea, muscle weakness, myalgia, sleeping disorders, memory issues, anxiety and depression [2–5]. These symptoms result in limitations in activities of daily living, a reduced working ability and a low quality of life [5–7].

Due to a lack of interventions that treat the condition itself, measures for symptomatic relief are of key importance. Accordingly, treatment guidelines recommend physiotherapy, complex rehabilitation interventions and advice on self-management [8–13]. Rehabilitation is defined as “a set of interventions designed to optimize functioning, health and wellbeing, and reduce disability in people with health conditions in interaction with their environment” [14]. Rehabilitation of PCC patients should be multidisciplinary including physical and psychological interventions [8, 15, 16]. The workforce may include physiotherapists, occupational therapists, psychologists and physicians with specialization in Physical and Rehabilitation Medicine (PRM) [9].

The WHO definition of PCC also includes all patients with persistent symptoms after severe infections and treatment in intensive care units. Some of these patients experience severe secondary neurological impairments of the peripheral and central nervous system ("Post intensive Care Syndrome, PICS") with cognitive, emotional and motor impairments that differ from the characteristic impairments of other subgroups of PCC patients [17]. Accordingly, these patients should be treated in line with guidelines on post-acute COVID-19 rehabilitation or PICS [17, 18].

Due to the new nature of PCC, rehabilitative measures were initially based on established interventions addressing PCC symptoms also found in other diseases such as fatigue or shortness of breath [19]. In the meantime, several systematic reviews [20–26] have indicated the effectiveness of some rehabilitation interventions. However, there is still an urgent need for additional clinical studies that evaluate different settings of rehabilitation in different subpopulations of PCC patients [23, 26].

In this article, we present a pilot observational study of a multidisciplinary bio-psycho-social rehabilitation (MBR) program with an emphasis on physiotherapy. As an introduction to this study, we present a review of physiotherapy in PCC patients.

### Review of physiotherapy in PCC patients

This review is based on 7 guidelines and clinical practice recommendations related to PCC [8–13, 27] and on 7 systematic reviews identified through a literature search on PubMed and Google Scholar [20–26]. The search strategy is presented in an online resource (reference 1).

### Physiotherapy

There is good evidence that physiotherapy treatments can improve fatigue, dyspnoea, physical deconditioning, muscle weakness, psychological function, and quality of life in patients with chronic obstructive pulmonary disease [28]. Since PCC patients share these symptoms, physiotherapy plays a key role in the treatment of patients with PCC. Depending on the symptoms, physiotherapy should be combined with neuropsychological trainings to improve cognition, health education to strengthen the ability of coping with fatigue and limitations in daily life and work, and psychiatric, psychological and psychosomatic therapy to reduce depressive symptoms and anxiety.

Before starting physical exercise training, physicians should screen for risks where physical training could cause an acute event or deterioration [27]. Therefore, a detailed medical history and physical examination including neurological, functional and psychological status is recommended for all patients [10]. In patients experiencing fatigue, other causes of fatigue such as thyroid disease, vitamin B12 deficiency, vitamin D deficiency, cardiac or hematologic/oncologic conditions should be excluded by respective laboratory tests [12]. Furthermore, it is important to thoroughly assess post-exertional symptom exacerbation (PESE), also known as post-exertional malaise or dysautonomia, before starting active treatment.

Patients with PESE report severe deterioration of symptoms like fatigue, cognitive dysfunction and exercise intolerance after physical or cognitive activity, which would not have caused any problem before the diseases onset [9]. PESE can be evaluated by standardized, validated self-reporting instruments [29]. However, PESE remains very subjective which is also true for other typical symptoms of post-COVID syndrome [30]. According to the WHO guideline, patients with PESE “are amenable to rehabilitation, their presence will require interventions to be modified in view of these diagnoses for rehabilitation to be safe” [9].

Shortness of breath, chest pain at low levels of exertion, a high resting pulse or a fast increase in pulse rate at low levels of exertion or a syncope can indicate a cardiac disease such as myocarditis [13]. Patients presenting with these symptoms need a cardiac examination, as recommended by guidelines [10]. Patients with myocarditis should avoid graded exercise therapy for three to six months.

Furthermore, autonomic dysfunction should be considered in patients who report intermittent sinus tachycardia, orthostatic tachycardia, vasovagal syncope or orthostatic hypotension [21]. Orthostatic tachycardia and orthostatic hypotension can be assessed using the active stand test [31].

In patients with dyspnoea, structural pulmonary diseases as pulmonary fibrosis should be considered. Additional diagnostic approaches may include functional tests at rest (especially pulmonary diffusion capacity, blood gas tests) and under stress (6-min walk test, ergospirometry), chest radiography and a blood test of D-Dimer [10]. However, PCC patients often report dyspnoea without abnormalities in lung function, imaging or cardiac examination [32].

Exertional oxygen desaturation should be evaluated using pulse oximetry before active physiotherapy and recommendations for sport [9]. It should be noted that exertional oxygen desaturation may not be recognized by the patient (silent hypoxemia). A decrease of 3% in oxygen desaturation after mild exertion is considered abnormal [33]. If exertional oxygen desaturation is observed, it should be ascertained that pulmonary and cardiac diagnostics have been carried out. If such diagnostics have not revealed any pathological findings, pulse oximetry during active physiotherapy is recommended to adjust the intensity of physical activity to prevent a decrease in oxygen saturation exceeding 3%.

Moreover, physicians should screen for mental disorders before recommending active physiotherapy. Patients with depression may experience a lack of motivation and reduced drive to engage in the recommended exercises. In these patients, psychiatric treatment such as psychotherapy should start prior or concurrently with active physical therapy.

#### Physiotherapy for specific symptoms and functional impairments

Pulmonary physiotherapy was initially developed for patients with chronic obstructive pulmonary disease (COPD). Restrictions in diaphragmatic function may contribute to the symptoms seen in COPD as well as after severe COVID-19 [34, 35]. It includes training of the inspiratory breathing muscles, training of the diaphragm and learning breathing techniques with deep and slow breathing as well as coughing exercises [36, 37]. This breathing pattern results in less energy expenditure, less airway irritation, reduced experience of breathlessness and less fatigue [13].

When treating patients with fatigue, it is important to distinguish between the symptoms of fatigue and the full picture of a chronic fatigue syndrome (CFS). The symptom fatigue is an overwhelming, debilitating, and sustained sense of exhaustion that decreases the ability to function and carry out daily activities [38]. In patients with symptom fatigue, there is good evidence for the effectiveness and safety of exercise therapy cognitive behavioural therapy [25, 39].

Patients with CFS show additional symptoms. The Nice guidelines define CFS with the four mandatory symptoms debilitating fatigability, PESE, unrefreshing sleep and cognitive difficulties [8]. However, in other case definitions, PESE is a frequent, but not mandatory symptom [30]. Patients with CFS require a multimodal therapy approach [9]. This includes skills training on energy conservation techniques such as pacing approaches, symptom-oriented physiotherapy and offering psychological support to the patients. In a multidisciplinary treatment concept, all health professionals should educate patients on the concept of pacing. The pacing concept has a positive effect on the number of PESE [40].

Regarding endurance and strength training with the principle of a fixed steady increase in physical activity (“graded exercise therapy = GRE”), there is an ongoing debate if GRE should be recommended or not in PCC patients with CFS [30]. The NICE guidelines recommend avoiding GRE for PCC patients with CFS [8]. In contrast, a recent systematic review concluded that patients with CFS may benefit from GRE [30]. According to our own experience, GRE with careful titration of intensity improves endurance in the majority of patients with PCC and fatigue. The severity of PESE should be taken into account when recommending for or against GRE. The Borg rating of the perceived exertion scale (PRE) can be applied for monitoring the exertion of patients [41].

PCC patients with physical deconditioning and muscle weakness can benefit from cardiopulmonary endurance training and muscle strength training according to the principal of GRE. Combined endurance training and strength training increases aerobic endurance, reduces fatigue and result in a better quality of life in patients with PCC [42].

If patients do not improve sufficiently with physiotherapy, the indication for complex rehabilitation interventions should be considered.

### Rehabilitation

The appropriate rehabilitation program for patients with PCC depends on the main symptoms and organ lesions. Patients with dyspnoea and reduced aerobic endurance can be referred to pulmonary rehabilitation, patients with neurological symptoms such as cognitive impairments or coordination disorders to neurological rehabilitation, patients with mental comorbidity or dysfunctional coping strategies to psychosomatic rehabilitation, and patients with cardiac impairment to cardiac rehabilitation. However, most patients with PCC exhibit deficits in various areas of physical and mental functions. Accordingly, a multidisciplinary approach that combines treatments for the different symptoms and functional limitations of the patients appears more suitable than an organ-specific approach for many PCC patients [16].

The functional limitations of PCC patients are often mentally stressful and can lead to social problems in the professional and family environment. Patients often feel themselves not properly understood, especially if they experience poor physical and mental health despite normal findings in the diagnostic procedures. Some patients react with social disengagement. This can result in further worsening of PCC symptoms such as fatigue and reduced physical and mental capacity. To break this vicious circle, multidisciplinary rehabilitation should address not only physical but also psychological and social factors following the bio-psycho-social model of functioning according to the International Classification of Functioning, Disability and Health (ICF) [43]. Initially, MBR programs were established in patients with chronic back pain. Clinical studies and systematic reviews demonstrated their effectiveness in improving pain and disability in patients with chronic low back pain and chronic neck pain and for a long period of time [44–46].

MBR programs in PCC patients combine active physiotherapy as described above with occupational therapy, psychological interventions, patient education and individual counselling on social and professional problems. A specific focus is on education on dealing with the disease and acquiring self-help strategies. In the following section, we describe the MBR program at the Ludwig-Maximilian-University Munich (LMU) University Hospital as an example of an MBR program.

### Multidisciplinary bio-psycho-social rehabilitation: a pilot study

#### Objective and study design

The objective of this observational study was to evaluate the feasibility of this PCC specific MBR program and to explore its effects on physical functioning. This study is part of the study “Continuous assessment of clinical course, outcome and resources in patients treated for Post-COVID—Post-COVID-Care”. It was approved by the institutional review board at the medical faculty of Ludwig Maximilian University Munich (Project Number 21–1165) and carried out in compliance with the Helsinki Declaration 2004. All participants provided informed consent prior to study participation.

#### Setting

The study was conducted at the day care clinic, PRM section of the Musculoskeletal University Center Munich (MUM), LMU University Hospital. Treatments were reimbursed by the German health insurance companies.

#### Participants and interdisciplinary assessment

This MBR program is integrated into the interdisciplinary PCC network of the LMU University Hospital. In this network, patients with severe PCC (Post Covid-19 Functional Status Scale (PCFS) score = 3 or 4; grades: minimum = 0; maximum = 4) [47] are initially seen in an interdisciplinary PCC outpatient clinic by an internist and a psychiatrist on referral from their general practitioner. Depending on the complaints, the PCC outpatient clinic refers patients to other departments such as pneumology, cardiology, neurology or psychiatry. Patients are referred to the PRM section if fatigue, severe physical deconditioning and muscle weakness are the main problems, but a persistent heart or lung lesion are excluded.

The PRM section of the MUM invites the referred patients to an interdisciplinary assessment. This is conducted by a specialist in Physical and Rehabilitation Medicine (PRM), a psychologist and either a physiotherapist or an occupational therapist. The assessment includes taking the medical history, appraisal of previous medical reports, patient-reported outcome measures (PROMs), standardized clinical tests and psychological exploration.

At the assessment (T0), fatigue is measured by the fatigue severity scale (FSS) [48], depressive symptoms and anxiety by the patient health questionnaire (PHQ-4) [49] and health-related quality of life by the 5-Level EuroQol questionnaire (EQ-5D-5L) and the EuroQol visual analogue scale (EQVAS) [50].

The FSS measures fatigue with nine questions [48]. Each question is scored on a scale from 1 to 7. The total score is the mean value. Patients scoring ≥ 4 are considered cases of fatigue. The PHQ-4 includes two items for depression and two items for anxiety [49]. Each response is scored on a scale from 0 to 3. Accordingly, the sum scores for each scale range from 0 to 6. Scores ≥ 3 are considered cases.

The EQ-5D-5L comprises the dimensions mobility, self-care, usual activity, pain/discomfort, and anxiety/depression [50]. The EQVAS records overall health on a scale ranging from 0 (= worst health status) to 100 (= best health status).

Physical endurance is assessed with the 6-min-walking test (6MWT) [51]. The mean walking distance for healthy adults is 581 ± 66.5 m for females and 608.7 ± 80.1 m for males [52]. The minimal important difference in patients with chronic obstructive pulmonary disease is 25 m [53]. After the 6MWT, physical exertion is asked on the PRE (Minimum = 0, Maximum = 10) [41]. Muscle strength of the lower extremities is tested with the 5-times sit-to-stand test (5STS) [54]. Both tests have been previously applied in several studies on PCC according to a recently published systematic review [26]. All assessors were trained physiotherapists or trained occupational therapists and followed a written manual.

Predefined inclusion criteria in the MBR program are a fatigue score ≥ 3 on the FSS, disease severity grade 3 on the PCFS scale, 300 to 700 m in the 6MWT, limitations of activities and participation, sufficient German language skills to follow the instructions in the MBR program and less than one hour travel time to the clinic. Patients were excluded from the MBR program if they had active myocarditis. The final decision for inclusion was based on the PROMs, the clinical tests and the global judgment by the treatment team that the patient would benefit from this treatment.

All participants of the MBR program from April 25, 2022 to March 28, 2023 who agreed and signed informed consent were included in the study.

#### Intervention

This intensive outpatient MBR program has specific characteristics that differ from typical inpatient rehabilitation programs in Germany. It is not organ or specialty-specific opposed to programs in rehabilitation clinics that are usually associated with a clinical specialty, i.e. pneumology. Treatment days alternate with free days to avoid overloading the patients. In addition, the patients can implement the content of the program in their home environment on their days off. All patients in this MBR program have the same functional status and experience fatigue. Furthermore, they have impairments in activities of daily living and participation including workability. The final decision on inclusion is made in an interdisciplinary assessment. This enables homogeneous treatment groups and facilitates an adaptation of the contents to individual capacity. This MBR program consists of only nine treatment days in a period of three weeks compared to usually 15 to 20 treatment days during three to four weeks in German rehabilitation clinics.

Patients are treated on three days per week (Monday-Wednesday-Friday or Tuesday-Thursday-Friday) for six to seven hours each day including breaks of 15 min after each treatment session and further breaks according to the individual needs of the patients. The lunch break is 1.5 h. During lunch break, patients have the option to lay down on beds. Table 1 provides an overview of the content, goals and structure of the program.

**Table 1:** Treatment program

Type of therapy	Content	Goals	Profession	Total frequency (duration), frequency per week
Assessment at entry	Physical examination, clinical tests, individual goal setting including the patients´ perspective	Evaluation of health status, defining treatment goals, and motivation of the patient	Specialist in PRM, physiotherapist or occupational therapist, psychologist	1 (90 min) Week 1: 1st day
Gym training (group)	Training with weight machines, treadmills, ergometer, and vibration plates	Improving muscle strength, endurance, postural control, coordination and balance	Physiotherapist	4 (60–90 min) Week 1: 1 Week 2: 2 Week 3: 1
Pulmonary physiotherapy (group)	Training inspiratory breathing muscles and diaphragm, learning breathing techniques with deep and slow breathing	Improve respiratory function and exercise tolerance, reduce chest pain and fatigue	Physiotherapist	8 (60 min) Week 1: 2 Week 2: 3 Week 3: 3
Physiotherapy (group)	Range of motion exercises, strength exercises with elastic bands, balance training, core strength exercises, mindful movement	Improve mobility, muscle strength, balance, physical capacity	Physiotherapist	
Occupational training (group)	Education in pacing, advice for structuring daily activities, olfactory sense training, instructions for home modification	Self-management of energy to avoid post-exertional symptom exacerbation, improve ability to perform daily tasks and working tasks, improve quality of life	Occupational therapist	6 (60—90 min) Week 1: 2 Week 2: 2 Week 3: 2
Cognitive training (individual)	Develop strategies to manage cognitive difficulties, computer-based cognitive training	Improve cognitive impairments (memory loss, difficulty concentrating, reduced attention span)	Occupational therapist	2 (60 min): Week 1 + 2 or week 2 + 3
Self-help techniques (group)	Instructions of self-help techniques such as warm packs, Kneipp hydrotherapy (repeated cold-water stimulations), self-massage techniques	Improving coping strategies, reducing fatigue, pain and discomfort	Medical massage therapist	6 (60 min) Week 1: 2 Week 2: 2 Week 3: 2
Pool exercises (group)	Aquatic exercises and swimming	Improving cardiorespiratory endurance and muscle strength, enhance mental relaxation	Physiotherapist, swimming trainer	6 (45 min) Week 1: 2 Week 2: 2 Week 3: 2
Psychological lessons (group)	Education in pacing and coping strategies with reduced physical capacity, encouraging joyful activities, learning to enjoy, teaching strategies to adhere to goals	Improving the ability to manage daily activities and workings tasks, improve ability to relax, reduce anxiety, improve inner drive and mood, improve fatigue, knowledge of strategies for adhering to goals	Psychologist	4 (60 min) Week 1: 1 Week 2: 1 Week 3: 1
Individual appointments with the PRM﻿ specialist	Individual evaluation of the course of symptoms and goal attainment; counselling on strategies to return to working life	Recognizing individual problems, adapting goals if necessary, developing strategies for lifestyle changes and returning to work	Specialist in PRM﻿ Psychologist	2 (30 min) Week 1 + 2 or week 2 + 3
Individual appointments with psychologist	Discussing patient-specific topics, such as psychosocial stressors, worries about the future, illness-related factors	Finding solutions together with the patient for problems in the vocational and social environment	Psychologist	2 (30 min): week 1 + 2 or week 2 + 3
Ward rounds (group)	Questioning each patient about symptoms and health problems	Recognizing individual problems of patients; documenting the course of symptoms	Specialist in PRM﻿	4 (30 min) Week 1: 1 Week 2: 2 Week 3: 1
Patient education (group)	Interactive presentation of background information; topics: up-to-date information on PCC treatment, fatigue management, work reintegration	Improving adherence to treatment by providing a theoretical background and up-to-date knowledge; helping patients to understand their health condition, providing recommendations for lifestyle modifications	Specialist in PRM﻿	3 (45 min) = week 1: 1 Week 2: 1 Week 3: 1
Group discussion with patients at the end of the week	Feedback round of patients and rehabilitation team	Consolidation of knowledge and intentions by reflection nf the last week; defining goals for next week; peer group support	Rehabilitation team, patient group	3 (30 min) = week 1: 1 Week 2: 1 Week 3: 1
Team meeting (group)	Discussion of all patients in the rehabilitation team	Recognizing problems of individual patients and defining solutions	Rehabilitation team	4 (30 min) Week 1: 2 Week 2: 1 Week 3: 1
Assessment at the end of the rehabilitation	Examination, clinical tests, individual setting of goals after the end of rehabilitation, development of an individual plan for action (e.g. therapy, work rehabilitation, lifestyle changes)	Evaluation of changes in the health status; motivation of the patient; maintaining and further improving benefits from the treatment; regaining workability	Specialist in PRM﻿, physiotherapist or occupational therapist, psychologist	1 (90 min) Week 3

*PRM*  Physical and Rehabilitation Medicine

The interdisciplinary treatment team consists of specialists in PRM, physiotherapists, occupational therapists, psychologists, medical masseurs and swimming trainers. The overall goals of the MBR program are improving symptoms and educating patients on coping with PCC. Furthermore, individual treatment goals on the levels of body functions, activities and participation according to the International Classification of Functioning, Disability and Health (ICF) [43] are defined on the first day of the MBR program together with the patient. The physician and psychologist discuss progress in these goals with the patients in the weekly personal appointments. The treatment team reviews the goals in group discussions at the end of each week and adjusts the goals if necessary.

The total group size of maximum of ten patients is divided into two subgroups of maximum 5 patients based on their physical fitness and level of fatigue. All active treatments take place in small subgroups. Furthermore, all patients have individual appointments with the psychologist and the physician each week.

The initial intensity of physical training is adapted to each patient’s individual physical performance according to the 6MWT and the reporting of PESE symptoms. Oxygen saturation during exercise is determined by pulse oximetry. If oxygen saturation is stable and patients show no signs of PESE, physical activity is gradually increased. If patients show signs of PESE, the intensity of physical training is reduced. Patients are encouraged to repeat some exercises at home on their days off with the aim of profound internalization of the exercises. However, they are asked to avoid overexertion. The treatment team provides information handouts to increase the sustainability of the program.

#### Data collection

At T0, patients completed questionnaires and the treatment team performed clinical tests as described above. At the beginning of the MBR program (T1) and at its end (T2), the clinical tests were repeated. Data were manually transferred from the forms to an Excel datasheet by a study nurse. Measures for data cleaning have been reported elsewhere [46].

#### Outcome measures

The PROMS at T0 included the FSS [48], the PHQ-4 [49], the EQ-5D-5L and EQVAS [50]. Physiological outcomes were assessed by the 6MWT [51], the Borg rating of PRE after the 6MWT [41] and the 5STS [54]. The primary outcome was the change of walking distance in the 6MWT. All data were prospectively collected.

#### Analyses

Criteria for the feasibility of this MBR were full participation (at least 8 of 9 treatment days) and at least stable physical endurance in the 6MWT. Reasons for early termination of the program associated with the treatment as well as for deterioration in physical endurance were retrospectively identified in the records of the patients. The effects of therapy on physical functioning were further explored by pre/post analyses of the 5STS and the PRE.

Statistical analyses were performed with the software package IBM SPSS Statistics 26 for windows. Changes in outcomes between T1 and T2 are reported by descriptive statistics. Statistical significance of changes was tested by Wilcoxon signed rank-sum tests for dependent samples because data were not normally distributed and the sample size was small. According to the concept of minimal clinical change in the 6MWT, patients were divided into those with improvement, those with no change and those with deterioration. Differences between patients with deterioration in the 6MWT compared to patients with stable or improved 6MWT were tested for statistical difference in sex and age by Fischer exact test and independent *t*-test respectively.

## Results

From April 25, 2022 to March 28, 2023, 70 patients were included in this MBR program. Of these, 37 had agreed to participate in the study. One terminated the MBR program after two treatment days due to worsening fatigue. One patient did not complete the treatment due to acute respiratory infection (no SARS-CoV-2 infection) and another patient due to an acute ankle injury outside the MBR program. Finally, one patient was excluded from the analysis of treatment effects because of missing two days of treatment due to severe fatigue.

The participant characteristics, comorbidities and baseline scores of the remaining 33 patients are presented in Table 2. The mean age was 42.8 ± 12.0 years. Females accounted for 72.7% of participants.

**Table 2:** Participant characteristics at assessment (*n* = 33)

Characteristic	yes/no (*N*) or mean (SD)	yes/no, valid %
Age, mean yrs. (SD)	42.8 (12.0)	
Female/Male	24/9	72.7/27.3
Comorbidities before SARS-COV-2		
Pulmonary diseases	11/22	33.3/66.7
Cardiovascular diseases	9/24	27.3/72.7
Thyroid diseases	10/23	30.3/69.7
Psychiatric diseases	6/27	18.2/81.8
Musculoskeletal and pain disorders	17/16	51.5/48.5
Other health conditions, yes/no	9/24	27.3/72.3
Depression (PHQ-4)		
> = 3/ < 3	11/22	33.7/67.3
Mean (SD)	2.03 (1.5)	
Anxiety (PHQ-4)		
> = 3/ < 3	11/22	33.7/67.3
Mean (SD)	1.94 (1.4)	
Fatigue (FSS)		
> = 4/ < = 4	29/5	87.9/12.1
Mean (SD)	5.7 (1.3)	
EQVAS, mean (SD)	47.0 (16.6)	
Education		
Basic or middle school (9–11y)/High school (> 11 y)	11/22	38.0/62.0
Employed/Non-Employed	28/5	84.8/15.3
Workability yes/no	0/28	0/100
Marital Status		
Living with partner/alone	20/13	60.6/29.4
Days PCR—Start MBR, mean (SD)	312 (162)	
Days ASS—Start MBR, mean (SD)	33 (26)	

The most frequent comorbidities before SARS-CoV-2 infection were musculoskeletal and pain disorders (51.5%), pulmonary diseases (33.3%), thyroid diseases (30.3%) and cardiovascular disease including arterial hypertension (27.3%). One-third of the patients showed depressive symptoms and one-third showed anxiety symptoms in the PHQ-4. The mean fatigue score in the FSS was 5.7. The EQVAS showed a much worse quality of life compared to the normal German population (47.0 vs. 71.6) [55]. All 28 employed persons were unable to work before the MBR program.

Table 3 presents the results of the clinical tests at T1 and T2. The 6MWT showed a numerical improvement of the mean score from 500.6 ± 97.4 m to 512.0 ± 87.4 m which was not statistically significant. The PRE was almost unchanged. According to the concept of the minimal important difference, the 6 MWT improved in 13 patients, remained stable in 14 patients and worsened in 6 patients. In the 5STS, the little numerical improvements were not significant.

**Table 3:** Outcome measures at entry to treatment and the end of treatment

	Entry		End	(SD)
	Mean	(SD)	Mean	
6-min walk, m	500.6	97.4	512.0^a^	87.4
PRE	4.35	2.17	4.53^a^	2.11
5 times sit-to-stand, s	14.0	6.3	13.1^a^	4.0

^a^All changes were not significant (*p*-values > 0.05)

### Reasons for early termination of the MBR program

In two patients, not completing the MBR program was probably associated with the MBR program itself. The only patient with less than 300 m in the 6MWT (exactly 258 m) terminated the MBR program after two treatment days. The other patient missed two days due to deterioration of fatigue. This patient had very severe fatigue at T0 (FSS-score = 6.8). However, he returned to the MBR program and had a stable walking distance in the 6MWT (T1: 506 m; T2; 501 m) and the exertion after the 6MWT was improved at T2 (T1: PRE = 5; T2: PRE = 3). Very low physical capacity and very high level of fatigue respectively may have caused premature termination of the program by these two patients.

### Causes for deterioration in physical endurance at the end of the MBR program

The comparison of the six patients with deterioration compared to patients with stable or improved 6MWT showed no significant differences in the sex distribution (83.3% vs. 70.4%) and age (47.3 ± 8.0 years vs. 41.7 ± 12.8 years).

Two of the six patients had considerably high fatigue scores of 6.7. Accordingly, symptom exacerbation due to overloading is a plausible cause for deterioration. In one patient, an iron deficiency (Fe 28 µg/dl; Ferritin 9 ng/dl) and in another patient Vitamin-D-deficiency (12.5 ng/ml) may have contributed to the unfavorable course of physical endurance. Iron supplementation and Vitamin D supplementation started at the beginning of the MBR program. One patient with pre-existing depression and panic attacks suffered severe panic attacks in the middle of the program and reported symptom exacerbation after this event. The sixth patient with deterioration in the 6MWT has reported a subjective overall improvement in physical endurance. However, pre-existing muscle pain had increased in the last week of the MBR. We suggest that this muscle pain may have caused the patient to walk more slowly in the 6MWT.

## Discussion

The results of this pilot study demonstrate the feasibility of an MBR program in a day-care setting for PCC patients who suffer from severe fatigue and a severely reduced functional status. This MBR distinguishes itself from other rehabilitation programs through its non-organ-specific multidisciplinary approach, the day-care setting, and the patient selection based on interdisciplinary assessment. Another unique aspect of this study is the detailed identification of reasons for therapy discontinuation or deterioration during the course of treatment.

In the present study, more than twice as many patients improved their physical endurance compared to the number of patients with deterioration. This confirms the WHO recommendation that active therapy is possible for the majority of patients with PCC and fatigue [9]. However, the results also showed that an active treatment program can lead to a worsening of physical capacity in some patients. The individual analysis of patients with deterioration suggest that pre-existing or de novo mental health conditions, Vitamin D deficiency and iron deficiency may be treated before the start of a rehabilitation intervention.

We found four previous observational studies on PCC rehabilitation that report a multidisciplinary treatment approach combining physiotherapy interventions, therapeutic education, and psychological sessions [16, 56–58]. All of them report significant improvements in the 6MWT with larger effects compared to our study. One possible reason for the larger effects is differences in the patient population because patients in the present study showed at baseline more severe fatigue, poor functional status and lower quality of life compared to the four other studies. Another cause for the small, non-significant improvement in our study is the relatively small number of nine treatment days. Indeed, based on the results of the present study, we have decided to extend the MBR program from nine treatment days in 3 weeks to 12 treatment days in 4 weeks.

The positive effects of exercise-based rehabilitation and respiratory training on physical functioning were also confirmed by a systematic review and meta-analysis based on 14 randomised controlled trials with a total of 1244 participants [26]. None of the interventions reported a multidisciplinary treatment approach. Patients had varying degrees of dyspnoea, but the frequency and severity of fatigue was not reported. The 6MWT was used in 7 trials. The mean walking distance improved by 36 m. These results suggest that patients with PCC whose main problem is dyspnoea but who do not have severe fatigue may benefit from physiotherapy and rehabilitation alone and may not need the more complex bio-psycho-social approach.

In this pilot study, PCC patients exhibited a greater impairment in their functional status and experienced more severe fatigue compared to earlier studies [16, 56–58]. The evaluation of their quality of life indicated a significantly greater impairment compared to other PCC subgroups [6, 57]. While our study demonstrated the feasibility and tolerability of a rehabilitation program emphasizing active physiotherapy in PCC patients with severe fatigue and low quality of life, further research is needed to provide evidence of its effectiveness.

The development of a generally acknowledged core set of outcome measures could facilitate comparisons across studies and allow performing meta-analyses. Important domains of outcome were defined in a sound international Delphi consensus study [59]. According to the authors of that study, consensus on the measurement instruments that are most appropriate for each outcome is the aim of a second phase of that project.

The present study has several limitations. Firstly, the patients included in this study were pre-selected through the interdisciplinary PCC outpatient of the LMU University Hospital, which may affect the generalizability of the results to other PCC populations. Secondly, the potential causes of a decline in physical endurance from the start to the end of the MBR in some patients were retrospectively collected from medical records. Consequently, it is possible that the associations between suspected causes and outcomes are coincidental rather than causal. Thirdly, the medium and long-term effects of the intervention were not investigated, as our primary focus was to promptly disseminate insights into multidisciplinary, non-organ-specific rehabilitation for severely affected PCC patients.

## Conclusions

To further improve the evidence on the effectiveness of physiotherapy and rehabilitation in PCC patients, future clinical trials should consider using control group designs, include higher numbers of participants and collect data about the health status before SARS-CoV-2-infection. A comprehensive description of the content of the intervention is essential to facilitate comparison between different interventions. Furthermore, it is important that adverse events and reasons for early termination are reported. Another important question is whether intensive, outpatient MBR programs like the one presented here, or inpatient treatment programs, show better outcomes.

Another important field of research is the evaluation of different treatment responses in patients that differ in the causes of PCC. Since many causes for PCC have been described, it can be assumed that patients with different causes for PCC require different therapeutic approaches. Linking basic research and clinical research could help to develop treatment strategies that best fit to specific subgroups of patients with PCC.

The high number of 169 registered studies in the WHO internal clinical trial registry platform in September 2022 in the field of rehabilitation of patients with PCC suggests further relevant evidence in the next future [60].

## Supplementary Information

Below is the link to the electronic supplementary material.

Supplementary file1 (DOCX 13 KB)

## Data Availability

Data are available upon reasonable request. The collected data cannot be shared publicly because the patients did not consent to the use of their data in a public repository. Access to any individual level patient data is not available.

## References

[1] World Health Organization 2022: Post COVID-19 condition (Long COVID). https://www.who.int/europe/news-room/fact-sheets/item/post-covid-19-condition. Accessed 11 June 2023

[2] Chaolin H, Lixue H, Yeming W, Xia Li, Ren Lili Gu, Xiaoying KL, Li G, Min L, Xing Z, Jianfeng L, Huang Zhenghui Tu, Shengjin ZY, Chen Li Xu, Decui LY, Caihong Li, Peng Lu, Yong Li, Wuxiang X, Dan C, Lianhan S, Fan Guohui Xu, Jiuyang WG, Ying W, Jingchuan Z, Chen W, Jianwei W, Dingyu Z, Bin C (2021) 6-month consequences of COVID-19 in patients discharged from hospital: a cohort study. The Lancet 397(10270):220–232. 10.1016/S0140-6736(20)32656-8 10.1016/S0140-6736(20)32656-8 PMC7833295 33428867

[3] Chen C, Haupert Spencer R, Zimmermann Lauren, Shi Xu, Fritsche Lars G, Mukherjee Bhramar (2022) Global Prevalence of Post-Coronavirus Disease 2019 (COVID-19) Condition or Long COVID: a meta-analysis and systematic review. J Infect Dis 226(9):1593–1607 35429399 10.1093/infdis/jiac136 PMC9047189

[4] Swarnakar R, Jenifa S, Wadhwa S (2022) Musculoskeletal complications in long COVID-19: a systematic review. World J Virol 11(6):485–495. 10.5501/wjv.v11.i6.485 36483107 10.5501/wjv.v11.i6.485 PMC9724204

[5] Lemhöfer C, Sturm C, Loudovici-Krug D, Best N, Gutenbrunner C (2021) The impact of Post-COVID-Syndrome on functioning—results from a community survey in patients after mild and moderate SARS-CoV-2-infections in Germany. J Occup Med Toxicol (London, England) 16(1):45. 10.1186/s12995-021-00337-9 10.1186/s12995-021-00337-9 PMC8495185 34620202

[6] Tabacof L, Tosto-Mancuso J, Wood J, Cortes M, Kontorovich A, McCarthy D, Rizk D, Rozanski G, Breyman E, Nasr L, Kellner C, Herrera JE, Putrino D (2022) Post-acute COVID-19 syndrome negatively impacts physical function, cognitive function, health-related quality of life, and participation. Am J Phys Med Rehabil 101(1):48–52. 10.1097/phm.0000000000001910 34686631 10.1097/PHM.0000000000001910 PMC8667685

[7] Christina L, Norman B, Christoph G, Dana L-K, Lidia T, Christian S (2021) Gefühlte und reale Arbeitsfähigkeit von Patient*innen mit Post-COVID Symptomatik nach mildem Akutverlauf: eine Analyse des Rehabilitation Needs Questionnaire (RehabNeQ). Physikalische Medizin, Rehabilitationsmedizin, Kurortmedizin 32(03):151–158. 10.1055/a-1674-8044

[8] National Institute for Health and Care Excellence (NICE) Scottish Intercollegiate Guidelines Network (SIGN) and Royal College of General Practitioners (RCGP) 2022: COVID-19 rapid guideline: managing the long-term effects of COVID-19. https://www.nice.org.uk/guidance/NG188. Accessed 11 June 2023

[9] World Health Organization 2023: Clinical management of COVID-19: Living guideline, 13 January 2023. https://www.who.int/publications/i/item/WHO-2019-nCoV-clinical-2023.1. Accessed 11 June 2023

[10] Koczulla AR, Ankermann T, Behrends U, Berlit P, Böing S, Brinkmann F, Franke C, Glöckl R, Gogoll C, Hummel T, Kronsbein J, Maibaum T, Peters EMJ, Pfeifer M, Platz T, Pletz M, Pongratz G, Powitz F, Rabe KF, Scheibenbogen C, Stallmach A, Stegbauer M, Wagner HO, Waller C, Wirtz H, Zeiher A, Zwick RH (2021) S1 Guideline Post-COVID/Long-COVID. Pneumologie (Stuttgart, Germany) 75(11):869–900. 10.1055/a-1551-9734 34474488 10.1055/a-1551-9734

[11] Teixido L, Andreeva E, Gartmann J, Lemhöfer C, Sturm C, Gutenbrunner C (2022) Ambulante Rehabilitative Versorgung von Patienten mit Long-COVID—eine leitlinienorientierte klinisch-praktische Handlungsempfehlung. Physikalische Medizin, Rehabilitationsmedizin, Kurortmedizin 32(06):365–376. 10.1055/a-1820-7396

[12] Centers for Disease Control and Prevention 2022: Post-COVID Conditions: Information for Healthcare Providers. Post-COVID Conditions: Information for Healthcare Providers (cdc.gov). Accessed 12 June 2023

[13] Greenhalgh T, Knight M, A’Court C, Buxton M, Husain L (2020) Management of post-acute covid-19 in primary care. BMJ (Clin Res ed) 370:m3026. 10.1136/bmj.m3026 10.1136/bmj.m3026 32784198

[14] World Health Organization 2023: Rehabilitation. https://www.who.int/news-room/fact-sheets/detail/rehabilitation. Accessed 11 June 2023

[15] Lovisa H, Levi R, Divanoglou A, Birberg-Thornberg U, Samuelsson K (2022) Seven domains of persisting problems after hospital-treated covid-19 indicate a need for a multiprofessional rehabilitation approach. J Rehabil Med. 10.2340/jrm.v54.2434 10.2340/jrm.v54.2434 PMC9422323 35678268

[16] Alexa K, Franziska E, Judit K, Volker K (2022) Nicht nur multimodal, sondern auch interdisziplinär: Ein Konzept für fächerübergreifende Zusammenarbeit in der Rehabilitation des Post-COVID-Syndroms. Psychother Psychosom Med Psychol 73(01):34–41. 10.1055/a-1838-3055 35605967 10.1055/a-1838-3055

[17] Platz T, Bergheim S, Berlit P, Dewey S, Dohle C, Fickenscher H, Grill E, Guha M, Köllner V, Kramer A, Reißhauer A, Schlitt A, Schultz K, Steimann M, Zeeb H (2023) S2k-Leitlinie SARS-CoV-2, COVID-19 und (Früh-) Rehabilitation – eine Kurzfassung mit allen Empfehlungen im Überblick. Rehabilitation (Stuttg) 62(2):76–85. 10.1055/a-1844-9984 35913083 10.1055/a-1844-9984

[18] Renner C, Jeitziner MM, Albert M, Brinkmann S, Diserens K, Dzialowski I, Heidler MD, Lück M, Nusser-Müller-Busch R, Sandor PS, Schäfer A, Scheffler B, Wallesch C, Zimmermann G, Nydahl P (2023) Guideline on multimodal rehabilitation for patients with post-intensive care syndrome. Crit Care 27(1):301. 10.1186/s13054-023-04569-5 37525219 10.1186/s13054-023-04569-5 PMC10392009

[19] Arienti C, Cordani C, Lazzarini SG, Del Furia MJ, Negrini S, Kiekens C (2022) Fatigue, post-exertional malaise and orthostatic intolerance: a map of Cochrane evidence relevant to rehabilitation for people with post COVID-19 condition. Eur J Phys Rehabil Med. 10.23736/s1973-9087.22.07802-9 36472558 10.23736/S1973-9087.22.07802-9 PMC10077961

[20] Fugazzaro S, Contri A, Esseroukh O, Kaleci S, Croci S, Massari M, Facciolongo NC, Besutti G, Iori M, Salvarani C, Costi S (2022) Rehabilitation Interventions for post-acute COVID-19 syndrome: a systematic review. Int J Environ Res Public Health. 10.3390/ijerph19095185 35564579 10.3390/ijerph19095185 PMC9104923

[21] Halabchi F, Selk-Ghaffari M, Tazesh B, Mahdaviani B (2022) The effect of exercise rehabilitation on COVID-19 outcomes: a systematic review of observationaAl and intervention studies. Sport Sci Health 18(4):1201–1219. 10.1007/s11332-022-00966-5 35789736 10.1007/s11332-022-00966-5 PMC9244056

[22] Hallek M, Adorjan K, Behrends U, Ertl G, Suttorp N, Lehmann C (2023) Post-COVID syndrome. Deutsches Arzteblatt international 120(4):48–55. 10.3238/arztebl.m2022.0409 36633452 10.3238/arztebl.m2022.0409 PMC10060997

[23] Chandan JS, Brown KR, Simms-Williams N, Bashir NZ, Camaradou J, Heining D, Turner GM, Rivera SC, Hotham R, Minhas S, Nirantharakumar K, Sivan M, Khunti K, Raindi D, Marwaha S, Hughes SE, McMullan C, Marshall T, Calvert MJ, Haroon S, Aiyegbusi OL (2023) Non-pharmacological therapies for post-viral syndromes, including Long Covid: a systematic review. Int J Environ Res Public Health. 10.3390/ijerph20043477 36834176 10.3390/ijerph20043477 PMC9967466

[24] Tamburlani M, Cuscito R, Servadio A, Galeoto G (2023) Effectiveness of respiratory rehabilitation in COVID-19s Post-acute phase: a systematic review. Healthcare (Basel, Switzerland). 10.3390/healthcare11081071 37107905 10.3390/healthcare11081071 PMC10137696

[25] de Sire Alessandro, Moggio L, Marotta N, Agostini F, Tasselli A, Ferrante VD, Curci C, Calafiore D, Ferraro F, Bernetti A, Taskiran OO, Ammendolia A (2022) Impact of rehabilitation on fatigue in post-COVID-19 patients: a systematic review and meta-analysis. Appl Sci 12(17):8593

[26] Pouliopoulou DV, Macdermid JC, Saunders E, Peters S, Brunton L, Miller E, Quinn KL, Pereira TV, Bobos P (2023) Rehabilitation interventions for physical capacity and quality of life in adults with post-COVID-19 condition: a systematic review and meta-analysis. JAMA Netw Open 6(9):e2333838. 10.1001/jamanetworkopen.2023.33838 37725376 10.1001/jamanetworkopen.2023.33838 PMC10509723

[27] DeMars J, Brown DA, Angelidis I, Jones F, McGuire F, O’Brien KK, Pemberton OD, Pemberton S, Tarrant R, Verduzco-Gutierrez M, Gross DP (2022) What is safe long COVID rehabilitation? J Occup Rehabil. 10.1007/s10926-022-10075-2 10.1007/s10926-022-10075-2 PMC9628454 36315323

[28] McCarthy B, Casey D, Devane D, Murphy K, Murphy E, Lacasse Y (2015) Pulmonary rehabilitation for chronic obstructive pulmonary disease. The Cochrane Database System Rev. 10.1002/14651858.CD003793.pub3 10.1002/14651858.CD003793.pub3 PMC10008021 25705944

[29] Cotler J, Holtzman C, Dudun C, Jason LA (2018) A brief questionnaire to assess post-exertional malaise. Diagnostics (Basel Switzerland). 10.3390/diagnostics8030066 30208578 10.3390/diagnostics8030066 PMC6165517

[30] White P, Abbey S, Angus B, Ball HA, Buchwald DS, Burness C, Carson AJ, Chalder T, Clauw DJ, Coebergh J, David AS, Dworetzky BA, Edwards MJ, Espay AJ, Etherington J, Fink P, Flottorp S, Garcin B, Garner P, Glasziou P, Hamilton W, Henningsen P, Hoeritzauer I, Husain M, Huys AML, Knoop H, Kroenke K, Lehn A, Levenson JL, Little P, Lloyd A, Madan I, van der Meer JWM, Miller A, Murphy M, Nazareth I, Perez DL, Phillips W, Reuber M, Rief W, Santhouse A, Serranova T, Sharpe M, Stanton B, Stewart DE, Stone J, Tinazzi M, Wade DT, Wessely SC, Wyller V, Zeman A (2023) Anomalies in the review process and interpretation of the evidence in the NICE guideline for chronic fatigue syndrome and myalgic encephalomyelitis. J Neurol Neurosurg Psychiatry. 10.1136/jnnp-2022-330463 37434321 10.1136/jnnp-2022-330463

[31] Dani M, Dirksen A, Taraborrelli P, Torocastro M, Panagopoulos D, Sutton R, Lim PB (2021) Autonomic dysfunction in “long COVID”: rationale, physiology and management strategies. Clin Med (Lond) 21(1):e63–e67. 10.7861/clinmed.2020-0896 33243837 10.7861/clinmed.2020-0896 PMC7850225

[32] Reuken PA, Franz M, Giszas B, Bleidorn J, Rachow T, Stallmach A (2022) Ancillary diagnostic testing in post-COVID patients. Dtsch Arztebl Int 119(31–32):544–545. 10.3238/arztebl.m2022.0216 36384925 10.3238/arztebl.m2022.0216 PMC9677543

[33] Núñez-Cortés R, Rivera-Lillo G, Arias-Campoverde M, Soto-García D, García-Palomera R, Torres-Castro R (2021) Use of sit-to-stand test to assess the physical capacity and exertional desaturation in patients post COVID-19. Chron Respir Dis 18:1479973121999205. 10.1177/1479973121999205 33645261 10.1177/1479973121999205 PMC7923980

[34] Boussuges A, Habert P, Chaumet G, Rouibah R, Delorme L, Menard A, Million M, Bartoli A, Guedj E, Gouitaa M, Zieleskiewicz L, Finance J, Coiffard B, Delliaux S, Brégeon F (2022) Diaphragm dysfunction after severe COVID-19: an ultrasound study. Front Med 9:949281. 10.3389/fmed.2022.949281 10.3389/fmed.2022.949281 PMC9448976 36091672

[35] Schulz A, Erbuth A, Boyko M, Vonderbank S, Gürleyen H, Gibis N, Bastian A (2022) Comparison of ultrasound measurements for diaphragmatic mobility, diaphragmatic thickness, and diaphragm thickening fraction with each other and with lung function in patients with chronic obstructive pulmonary disease. Int J Chron Obstruct Pulmon Dis 17:2217–2227. 10.2147/copd.S375956 36118281 10.2147/COPD.S375956 PMC9480595

[36] Del Corral T, Fabero-Garrido R, Plaza-Manzano G, Fernández-de-Las-Peñas C, Navarro-Santana M, López-de-Uralde-Villanueva I (2023) Home-based respiratory muscle training on quality of life and exercise tolerance in long-term post-COVID-19: randomized controlled trial. Ann Phys Rehabil Med 66(1):101709. 10.1016/j.rehab.2022.101709 36191860 10.1016/j.rehab.2022.101709 PMC9708524

[37] Jimeno-Almazán A, Buendía-Romero Á, Martínez-Cava A, Franco-López F, Sánchez-Alcaraz Bernardino J, Courel-Ibáñez J, Pallarés JG (2023) Effects of a concurrent training, respiratory muscle exercise, and self-management recommendations on recovery from post-COVID-19 conditions: the RECOVE trial. Long-Term Recov SARS-CoV-2 (COVID-19) 134(1):95–104. 10.1152/japplphysiol.00489.2022 10.1152/japplphysiol.00489.2022 PMC9829459 36476156

[38] Matura LA, Malone S, Jaime-Lara R, Riegel B (2018) A systematic review of biological mechanisms of fatigue in chronic illness. Biol Res Nurs 20(4):410–421. 10.1177/1099800418764326 29540066 10.1177/1099800418764326 PMC6346311

[39] Rosenthal TC, Majeroni BA, Pretorius R, Malik K (2008) Fatigue: an overview. Am Fam Physician 78(10):1173–1179 19035066

[40] Parker M, Hannah BS, Flannery T, Tarrant R, Shardha J, Bannister R, Ross D, Halpin S, Greenwood DC, Sivan M (2023) Effect of using a structured pacing protocol on post-exertional symptom exacerbation and health status in a longitudinal cohort with the post-COVID-19 syndrome. J Med Virol 95(1):e28373. 10.1002/jmv.28373 36461167 10.1002/jmv.28373 PMC9878088

[41] Borg GA (1982) Psychophysical bases of perceived exertion. Med Sci Sports Exerc 14(5):377–381 7154893

[42] Araújo BTS, Barros A, Nunes DTX, Remígio de Aguiar MI, Mastroianni VW, de Souza JAF, Fernades J, Campos SL, Brandão DC, Dornelas de Andrade A (2023) Effects of continuous aerobic training associated with resistance training on maximal and submaximal exercise tolerance, fatigue, and quality of life of patients post-COVID-19. Physiother Res Int 28(1):e1972. 10.1002/pri.1972 36088642 10.1002/pri.1972 PMC9539049

[43] World Health Organization: International Classification of Functioning, Disability and Health: ICF. Geneva: WHO; 2001. International Classification of Functioning, Disability and Health (ICF) (who.int). Accessed 12 June 2023

[44] Kamper SJ, Apeldoorn AT, Chiarotto A, Smeets RJ, Ostelo RW, Guzman J, van Tulder MW (2014) Multidisciplinary biopsychosocial rehabilitation for chronic low back pain. The Cochrane Database Syst Rev. 10.1002/14651858.CD000963.pub3 25180773 10.1002/14651858.CD000963.pub3 PMC10945502

[45] Letzel J, Angst F, Weigl MB (2019) Multidisciplinary biopsychosocial rehabilitation in chronic neck pain: a naturalistic prospective cohort study with intraindividual control of effects and 12-month follow-up. Eur J Phys Rehabil Med 55(5):665–675. 10.23736/s1973-9087.18.05348-0 30468363 10.23736/S1973-9087.18.05348-0

[46] Ochsenkuehn FR, Crispin A, Weigl MB (2022) Chronic low back pain: a prospective study with 4 to 15 years follow-up after a multidisciplinary biopsychosocial rehabilitation program. BMC Musculoskelet Disord 23(1):977. 10.1186/s12891-022-05963-w 36369042 10.1186/s12891-022-05963-w PMC9650911

[47] Frederikus AK, Gudula JAMB, Stefano B, Endres M, Geelhoed JJM, Knauss S, Rezek SA, Spruit MA, Jörg V, Bob S (2020) The Post-COVID-19 Functional Status scale: a tool to measure functional status over time after COVID-19. Eur Resp J 56(1):2001494. 10.1183/13993003.01494-2020 10.1183/13993003.01494-2020 PMC7236834 32398306

[48] Krupp LB, LaRocca NG, Muir-Nash J, Steinberg AD (1989) The fatigue severity scale: application to patients with multiple sclerosis and systemic lupus erythematosus. Arch Neurol 46(10):1121–1123. 10.1001/archneur.1989.00520460115022 2803071 10.1001/archneur.1989.00520460115022

[49] Kroenke K, Spitzer RL, Williams JB, Lowe B (2009) An ultra-brief screening scale for anxiety and depression: the PHQ-4. Psychosomatics 50(6):613–621. 10.1176/appi.psy.50.6.613 19996233 10.1176/appi.psy.50.6.613

[50] Herdman M, Gudex C, Lloyd A, Janssen M, Kind P, Parkin D, Bonsel G, Badia X (2011) Development and preliminary testing of the new five-level version of EQ-5D (EQ-5D-5L). Qual Life Res 20(10):1727–1736. 10.1007/s11136-011-9903-x 21479777 10.1007/s11136-011-9903-x PMC3220807

[51] Guyatt GH, Sullivan MJ, Thompson PJ, Fallen EL, Pugsley SO, Taylor DW, Berman LB (1985) The 6-minute walk: a new measure of exercise capacity in patients with chronic heart failure. Can Med Assoc J 132(8):919–923 3978515 PMC1345899

[52] Cazzoletti L, Zanolin ME, Dorelli G, Ferrari P, Dalle CLG, Crisafulli E, Alemayohu MA, Olivieri M, Verlato G, Ferrari M (2022) Six-minute walk distance in healthy subjects: reference standards from a general population sample. Respir Res 23(1):83. 10.1186/s12931-022-02003-y 35382813 10.1186/s12931-022-02003-y PMC8985335

[53] Holland AE, Hill CJ, Rasekaba T, Lee A, Naughton MT, McDonald CF (2010) Updating the minimal important difference for six-minute walk distance in patients with chronic obstructive pulmonary disease. Arch Phys Med Rehabil 91(2):221–225. 10.1016/j.apmr.2009.10.017 20159125 10.1016/j.apmr.2009.10.017

[54] Whitney SL, Wrisley DM, Marchetti GF, Gee MA, Redfern MS, Furman JM (2005) Clinical measurement of sit-to-stand performance in people with balance disorders: validity of data for the Five-Times-Sit-to-Stand Test. Phys Ther 85(10):1034–1045 16180952

[55] Grochtdreis T, Dams J, König HH, Konnopka A (2019) Health-related quality of life measured with the EQ-5D-5L: estimation of normative index values based on a representative German population sample and value set. Eur J Health Econ 20(6):933–944. 10.1007/s10198-019-01054-1 31030292 10.1007/s10198-019-01054-1

[56] Hayden MC, Limbach M, Schuler M, Merkl S, Schwarzl G, Jakab K, Nowak D, Schultz K (2021) Effectiveness of a three-week inpatient pulmonary rehabilitation program for patients after COVID-19: a prospective observational study. Int J Environ Res Public Health. 10.3390/ijerph18179001 34501596 10.3390/ijerph18179001 PMC8430843

[57] Nopp S, Moik F, Klok FA, Gattinger D, Petrovic M, Vonbank K, Koczulla AR, Ay C, Zwick RH (2022) Outpatient pulmonary rehabilitation in patients with long COVID improves exercise capacity, functional status, dyspnea, fatigue, and quality of life. Respiration 101(6):593–601. 10.1159/000522118 35203084 10.1159/000522118 PMC9059007

[58] Ostrowska M, Rzepka-Cholasińska A, Pietrzykowski Ł, Michalski P, Kosobucka-Ozdoba A, Jasiewicz M, Kasprzak M, Kryś J, Kubica A (2023) Effects of multidisciplinary rehabilitation program in patients with long COVID-19: Post-COVID-19 rehabilitation (PCR SIRIO 8) study. J Clin Med 12(2):420. 10.3390/jcm12020420 36675349 10.3390/jcm12020420 PMC9864838

[59] Munblit D, Nicholson T, Akrami A, Apfelbacher C, Chen J, De Groote W, Diaz JV, Gorst SL, Harman N, Kokorina A, Olliaro P, Parr C, Preller J, Schiess N, Schmitt J, Seylanova N, Simpson F, Tong A, Needham DM, Williamson PR, Guekht A, Semple Malcolm CG, Warner JO, Sigfrid L, Scott JT, Dunn GA, Genuneit J, Buonsenso D, Sivan M, Siegerink B, Klok FA, Avdeev S, Stavropoulou C, Michelen M, Aiyegbusi OL, Calvert M, Hughes SE, Haroon S, Fregonese L, Carson G, Knauss S, O’Hara M, Marshall J, Herridge M, Murthy S, Vos T, Wulf HS, Parker A, O’Brien KK, Lerner A, Chevinsky JR, Unger ER, Eisinger RW, Hough CL, Saydah S, Frontera JA, Rosa RG, Cao B, Bhatnagar S, Thiruvengadam R, Seahwag A, Bouraoui A, Van KM, Dua T, Relan P, Soriano OJ (2022) A core outcome set for post-COVID-19 condition in adults for use in clinical practice and research: an international Delphi consensus study. The Lancet Resp Med 10(7):715–724. 10.1016/S2213-2600(22)00169-2 10.1016/S2213-2600(22)00169-2 PMC9197249 35714658

[60] Fawzy NA, Abou SB, Taha RM, Arabi TZ, Sabbah BN, Alkodaymi MS, Omrani OA, Makhzoum T, Almahfoudh NE, Al-Hammad QA, Hejazi W, Obeidat Y, Osman N, Al-Kattan KM, Berbari EF, Tleyjeh IM (2023) A systematic review of trials currently investigating therapeutic modalities for post-acute COVID-19 syndrome and registered on WHO International Clinical Trials Platform. Clin Microbiol Infect 29(5):570–577. 10.1016/j.cmi.2023.01.007 36642173 10.1016/j.cmi.2023.01.007 PMC9837206

