# Supplementary material for: Multidisciplinary rehabilitation with a focus on physiotherapy in patients with Post Covid19 condition: an observational pilot study
Source: Eur Arch Psychiatry Clin Neurosci. 2024 Jan 17;274(8):2003–14. doi: 10.1007/s00406-023-01747-y (PMC11579062; doi:10.1007/s00406-023-01747-y)
Supplement: Supplementary file 1 — Supplementary file1 (DOCX 13 KB) [file 406_2023_1747_MOESM1_ESM.docx]

**Online resource 1**

**Search strategy**

1. Seven guidelines and clinical practice recommendations for the treatment of PCC were identified by the authors expert knowledge [8-13, 27]

2. Systematic literature search in Pubmed and Google-Scholar using the following search terms on 10th February 2023: (Rehabilitation OR Exercise OR „Exercise therapy" OR "Physical Modalities" OR „Mind-Body Therapies" OR Physiotherapy). In Pubmed, we added the filters LitCLONGCOVID, systematic reviews, 5 years. In google scholar, we added AND “Post-Covid” AND “Systematic review” and screened the first 30 hits. The pubmed search resulted in 3 eligible reviews [20, 21]. From the google scholar search, 3 reviews were added from the first 30 hits [23, 24, 25].

3. One review was added by the authors expert knowledge [22].

4. One additional review was identified at the time of preparing the revised manuscript [26].

5. Clinical studies were added to the literature based on the authors' expert knowledge or citations of the guidelines and reviews that were identified.
